# The Etiology and Molecular Mechanism Underlying Smooth Muscle Phenotype Switching in Intimal Hyperplasia of Vein Graft and the Regulatory Role of microRNAs

**DOI:** 10.3389/fcvm.2022.935054

**Published:** 2022-07-28

**Authors:** Dengshen Zhang, Yiran Cao, Daxing Liu, Jian Zhang, Yingqiang Guo

**Affiliations:** ^1^Department of Cardiovascular Surgery, Affiliated Hospital of Zunyi Medical University, Zunyi, China; ^2^Department of Cardiovascular Surgery, West China Hospital, Sichuan University, Chengdu, China

**Keywords:** miRNAs, VSMCs, phenotypic transformation, intimal hyperplasia, vein graft

## Abstract

Mounting evidence suggests that the phenotypic transformation of venous smooth muscle cells (SMCs) from differentiated (contractile) to dedifferentiated (proliferative and migratory) phenotypes causes excessive proliferation and further migration to the intima leading to intimal hyperplasia, which represents one of the key pathophysiological mechanisms of vein graft restenosis. In recent years, numerous miRNAs have been identified as specific phenotypic regulators of vascular SMCs (VSMCs), which play a vital role in intimal hyperplasia in vein grafts. The review sought to provide a comprehensive overview of the etiology of intimal hyperplasia, factors affecting the phenotypic transformation of VSMCs in vein graft, and molecular mechanisms of miRNAs involved in SMCs phenotypic modulation in intimal hyperplasia of vein graft reported in recent years.

## Introduction

Coronary artery disease (CAD) is well-established as a significant threat to human health. With the rapid development of society and changes in lifestyle, the incidence of CAD has rapidly escalated in recent years, with the total number of CAD patients in China estimated to be more than 11 million (1). Coronary artery bypass grafting (CABG) surgery is a surgical intervention for CAD, and the grafted vessels often consist of the internal mammary artery, radial artery, and human saphenous vein (HSV). Arterial grafts are often inadequate for recanalization of CABG due to the limitations for harvesting, number and length. Therefore, HSV remains the most widely used for CABG in complex cases with multivessel disease since it is easier to manipulate. Unfortunately, the incidence of vein graft restenosis after CABG is high, with vein graft failure rates of up to 50% within 10 years of surgery, which seriously affects the long-term outcome of CABG surgery (2, 3). An increasing body of evidence suggests that intimal hyperplasia plays a major part in the pathophysiology of vein graft restenosis (4, 5). Indeed, exploring the molecular mechanisms underlying the occurrence of intimal hyperplasia in vein graft is of significant concern in CAD prevention and treatment.

Studies have shown that phenotypic transformation of VSMCs causing excessive proliferation of VSMCs and further migration to the intima is one of the primary pathological mechanisms that contribute to intimal hyperplasia in vein grafts (6–8). Therefore, there is an urgent need to identify novel molecules that can effectively control the phenotypic transformation of VSMCs. To date, miRNAs have been identified as phenotypic regulators of VSMCs with significant specificity, and most of them are endogenous small interfering RNAs with potential clinical applications.

### The Phenotypes of Vascular Smooth Muscle Cells (VSMCs) and Intimal Hyperplasia in Vein Grafts

**FIGURE 1:**
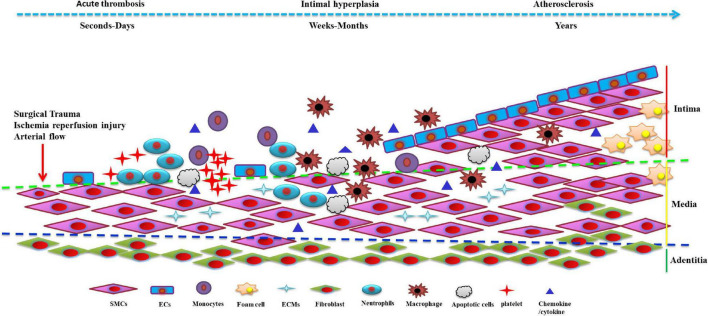
The phenotype of venous smooth muscle cells (SMCs) in the development of vein graft restenosis: Stimulated by various injuries, pathological factors and stress alterations, venous SMCs can transform from a differentiated to a dedifferentiated state, accompanied by a decrease in the expression of the above-mentioned differentiation-specific markers. Venous vascular smooth muscle cells (VSMCs) secrete many extracellular matrix (ECMs) and cytokines, causing excessive proliferation and further migration to the intima leading to intimal hyperplasia resulting in vein graft restenosis. SMCs, smooth muscle cells; ECs, endothelial cells; ECMs, extracellular matrix.

Vein graft restenosis is generally divided into 3 stages: early thrombosis, early to mid-stage intimal hyperplasia and accelerated atherosclerosis, with each of these stages having distinct properties while interweaving and overlapping to promote pathogenesis (4, 5). It is well-established that VSMCs control the vascular microenvironment and secure vascular morphology and function from the intima-media of the vessel wall. VSMCs can switch between differentiated (contractile) and dedifferentiated (synthesis) phenotypes depending on their functions. The dedifferentiated phenotype exhibits a higher proliferative, migratory and synthetic rate of extracellular matrixs (ECMs), thereby being termed the proliferative-migratory or synthetic phenotype. Differentiated VSMCs are marked by the expression of smooth muscle α-actin (SMα-actin), smooth muscle 22α protein (SM22α), myosin heavy chain (SM-MHC), calponin and other molecules, which play an essential role in regulating vascular physiological status such as vasodilation and contraction (9). Dedifferentiated VSMCs often express proteins such as osteopontin (OPN) and proliferating cell nuclear antigen (PCNA) in large amounts, acknowledged as markers of dedifferentiated VSMCs (10, 11). VSMCs within the vein exhibit a quiescent phenotype in their natural state, equipped with contractile and low proliferative and migratory capacities. VSMCs originate from the mesoderm with prodigious plasticity (12, 13). When various injuries and pathological factors stimulate vessels, VSMCs can transform from a differentiated to a dedifferentiated state, accompanied by decreased expression of the above-mentioned differentiation-specific markers and augmented expression of dedifferentiated molecules. Meanwhile, VSMCs become abnormally active in proliferation and migration and secrete many ECMs and cytokines. This process is referred to as the phenotypic switch of VSMCs (14–16). There is ample evidence suggesting that excessive proliferation and migration of dedifferentiated VSMCs and the synthesis of excessive ECMs participate in the pathogenesis of intimal hyperplasia in vein grafts (Figure 1) (6–8).

### Etiology of Intimal Hyperplasia in Vein Graft and Phenotypes of Vascular Smooth Muscle Cells (VSMCs)

Intimal hyperplasia in vein graft results from the interplay of multiple factors. The widely acknowledged causes of intimal hyperplasia in vein grafts are injury from surgery, ischemia-reperfusion injury, and stress alterations in venous arterialization, which are associated with dysfunction and phenotypic switch of VSMCs.

#### Vascular Injury and Phenotypic Switch of Vascular Smooth Muscle Cells (VSMCs) due to Surgical Operations

In patients requiring CABG, procedures such as manipulating, harvesting, examining and dilating the HSV can cause vein injury. The endothelium impedes platelet-derived growth factor production (17), suppresses inflammation (18), prevents platelet aggregation (19, 20), and inhibits VSMCs activity (21). Hence, the integrity of the endothelial monolayer is the premise for preserving its function. When the endothelium is damaged, the subendothelial matrix components become exposed to blood, which induces platelet aggregation and adhesion of inflammatory cells, resulting in the activation of coagulation pathways and acute thrombus formation (22). Moreover, the release of high amounts of tissue factors causes altered behavior of VSMCs, which provides the basis for later restenosis (4). Damage to the endothelium also jeopardizes nitric oxide (NO) secretion. Endothelial-derived NO inhibits the synthesis of tissue factors and the expression of metalloproteinases (MMPs) (23, 24). On the one hand, tissue factor activates VSMCs, driving intimal hyperplasia in the grafted vein (4, 25). On the other hand, MMPs give rise to ECMs degradation and remodeling, facilitating the migration of VSMCs across the ECMs layer to the intimal, exacerbating intimal hyperplasia of vein graft (26). Additionally, vascular injury promotes the synthesis and expression of platelet-derived growth factor-BB (PDGF-BB), transforming growth factor-β (TGF-β), fibroblast growth factor (bFGF), insulin growth factor (IGF-1), angiotensin (Ang-II), tumor necrosis factor-α (TNF-α), and interleukin-1β (IL-1β), driving the activation and altered behaviors of VSMCs, enhancing the proliferative and migratory capacity, eventually leading to intimal hyperplasia in vein graft (25–28).

#### Ischemia-Reperfusion Injury and Phenotypic Switch of Vascular Smooth Muscle Cells (VSMCs)

During HSV harvesting and transplantation into the coronary artery, the vein graft vessels are in an ischemic-hypoxic state. After surgery, superoxides produced by ischemia-reperfusion injury represent one of the mechanisms by which IH occurs in vein grafts. Superoxides reduce NO synthesis, promote MMPs expression, and accelerate the phenotypic switch of VSMCs (29, 30). During the harvesting of the HSV, the traditional operation requires stripping the perivascular tissue and fat of the venous epithelium, which disconnects and damages the vasa vasorum. Accordingly, this procedure is an essential element leading to an ischemic and hypoxic state in vein grafts (31). In addition, we suggest that the ischemic-hypoxic phase of vessels should include angiogenesis of vasa vasorum in grafted veins. A favorable long-term patency rate could be reached by applying the “no-touch” technique for HSV harvesting *via* preoperative ultrasound localization, which preserves as much as possible vasa vasorum around the HSV and reduces ischemic-hypoxic injury (32). Moreover, studies have demonstrated that hypoxic injury can drive remodeling and phenotypic switch of VSMCs, triggering their early abnormal proliferation (33). Taken together, the above findings suggest that the occurrence of intimal hyperplasia in grafted veins may be due to the phenotypic switch of VSMCs caused by ischemic-hypoxic injury.

#### Stress Alterations in Venous Arterialization and Phenotypic Switch of Vascular Smooth Muscle Cells (VSMCs)

It is well-established that blood flow yields contrasting stress stimuli on the vessel wall (34). The mechanical milieu maintains vascular functional homeostasis and serves as a predisposing contributor to many cardiovascular diseases. The vessel wall bears complex stress, mainly subject to shearing stress along the direction of blood flow and circumferential stress from blood pulsation (35). VSMCs play a crucial role in resisting challenges in the vascular mechanical microenvironment and regulating vascular function (36). The shear stress and circumferential stress of venous flow in the physiological state are 1.5–6 dyn/cm^2^ and 1%, respectively, while the arteries are about 20–70 dyn/cm^2^ and 10–15% (8, 37–40). It has been shown that vein grafts are subjected to chronic shear and circumferential stresses in the arterial circulation after implantation (37–39). The neointima forms approximately 2 weeks after surgery, indicating complete repair of venous ECs, but intimal hyperplasia still occurs, suggesting the lesion is still present. Besides, stress alterations persist, justifying its role in vein graft restenosis. Morphological, genetic and metabolic differences between vein and artery lead to distinct responses to stress (40). The HSV is infrequently stenosed unless grafted in an arterial setting. When used as a graft vessel, the internal thoracic artery exhibits patency at 10 years in 90% of cases, whereas most veins fail to stay patent for the same period (41). These studies substantiate that hemodynamic alterations participate in vein graft intimal hyperplasia. When a vein graft is connected to an artery, it sustains stress from the arterial flow. ECs in the vein graft undergo high-flow shear washout and dislodgement, and this stress subsequently stimulates VSMCs in the grafted vein are directly exposed to a circumferential arterial stretch, which can fuel the phenotypic transformation of VSMCs (36–39), translating into massive proliferation and migration of VSMCs, and leading to intimal hyperplasia of grafted vein (6–8). Intimal hyperplasia formation accelerates atherosclerosis as a prelude to vein graft occlusion.

In summary, the three factors described above do not exist alone and often interact to promote intimal hyperplasia of vein grafts. During CABG, the direct injury during vein harvesting, subsequent ischemia-reperfusion injury, and sustained stress alterations due to vein arterialization propel function remodeling and phenotypic switch of VSMCs into a dedifferentiated state with excessive replication, migration to the intima, and synthesis of large amounts of ECMs, conducive to the development of intimal hyperplasia in vein grafts (25, 32, 33, 39). Accordingly, unveiling the molecular pathogenesis of phenotypic transformation of VSMCs in grafted veins and identifying prevention and treatment strategies are important to minimize venous graft stenosis after CABG.

### Molecular Mechanism of Intimal Hyperplasia in Vein Graft and Phenotypic Switch of Vascular Smooth Muscle Cells (VSMCs)

#### Tissue Factor and Growth Factor and Phenotypic Switch of Vascular Smooth Muscle Cells (VSMCs)

Many growth factors and cytokines such as PDGF-BB, TGF-β, bFGF, and IGF-1 can promote VSMCs dedifferentiation (42–45). PDGF-BB is a pro-cytokine that stimulates cell division and is the most potent inducer of phenotypic transformation of VSMCs investigated (46, 47). PDGF-BB earned its name for the secretion of platelet α granules. Current evidence suggests that PDGF-BB can be synthesized and secreted by VSMCs, inflammatory cells, fibroblasts and ECs in response to tissue injury or stimuli (48, 49). Studies have shown that PDGF-BB levels are firmly correlated with the degree of intimal hyperplasia in grafted veins (50, 51). PDGF-BB synthesis is increased in vein grafts and remains stable in arterial grafts (48). Interestingly, it has been shown that blocking PDGF-BB inhibits intimal hyperplasia in HSV (52), and cultures have shown that PDGF-BB induces replication and migration of VSMCs of HSV but rarely affects VSMCs in internal thoracic arteries (53, 54). Dong et al. found that PDGF-BB involving ERK1/2 signal pathway activation could upregulate vascular cell adhesion molecule-1(VCAM-1) level, which led to the phenotypic transformation of VSMCs and exacerbated intimal hyperplasia in balloon injury of rat carotid arteries (55). Xiang et al. found that PDGF-BB could regulate the expression of the inflammatory and adhesion factor VCAM-1 *via* the JAK2/STAT3 signal pathway, which affected intimal hyperplasia in grafted veins (25). For this reason, PDGF-BB has become the most widely used method for inducing phenotypic switch of VSMCs and harming vascular cells.

#### Metalloproteinases, Extracellular Matrix and Phenotypic Switch of Vascular Smooth Muscle Cells (VSMCs)

It has been shown that MMPs affect the synthesis and degradation of ECMs such as collagen fibrils and glycoproteins under physiological conditions. During vascular remodeling, ECMs can be degraded by MMPs and remodeled to facilitate VSMCs migration through the ECMs layer to the intima, causing vascular intimal hyperplasia (56, 57). MMPs are widely expressed during intimal hyperplasia of grafted veins, and MMP-2/9 could promote VSMCs migration to the HSV, which can be inhibited by MMP-2/9 knockdown (58–60). Clinical studies have shown that MMP-2 expression in vein grafts is associated with CABG prognosis (61). Moreover, tissue inhibitors of metalloproteinases (TIMPs) alleviate intimal hyperplasia in vein graft by reducing MMPs expression and thus reducing VSMCs migration (62). The aforementioned studies provide important insights into the functions of MMPs and ECMs in the phenotypic transformation of VSMCs.

#### Inflammatory Factors and Phenotypic Switch of Vascular Smooth Muscle Cells (VSMCs)

The inflammatory cascade is vital for triggering vein graft intimal hyperplasia, which participates in the pathogenesis of late stages of vein graft atherosclerosis (63, 64). Increased permeability and disrupted function of ECs drive leukocyte activation and aggregation, followed by secretion of multiple chemokines and cytokines, which subsequently promote the activation of inflammatory cells. The knock-on effect induces VSMCs to proliferate and migrate (65, 66). Interestingly, in an external jugular vein-abdominal aortic graft rat model, nuclear transcription factor kappa B (NF-κB) remained active at high levels 4 weeks after surgery. After siRNA silenced NF-κB, vein graft intimal hyperplasia was significantly ameliorated (67). Since NF-κB activation is the core of inflammation, it is reasonable to speculate that inhibiting the inflammatory cascade could stabilize the differentiated phenotype of venous VSMCs to exert an anti- intimal hyperplasia effect. NF-κB is a critical transcriptional regulator in the transcription of inflammatory factors such as IL-1, IL-6, TNF-α, the C-C motif chemokine ligand 2 (CCL2/MCP-1), ICAM-1 and VCAM-1 (68, 69). These elements indicate that cytokines, chemokines and adhesion molecules mediate the recruitment and infiltration of inflammatory cells into the grafted vessel wall. CCL2/MCP-1 is an important mediator of vascular inflammation and the most potent chemotactic factor for monocytes. After vein grafting, the CCL2/MCP-1 level increases markedly and persists for several weeks (70). Blocking CCL2/MCP-1 and its receptor can inhibit monocyte adhesion in the vessel wall, attenuating artery intimal hyperplasia and atherosclerosis (71). Knocking down the expression of CCL2/MCP-1 by siRNA can dramatically reduce the proliferation and migration of VSMCs (72). Moreover, it has been shown that VCAM-1 interacts with integrin α4β1 on the leukocyte surface and triggers inflammatory cascade signaling (73). VCAM-1 promotes VSMCs to proliferate and migrate, and restricting VCAM-1 expression was found to attenuate intimal hyperplasia in a rat model of carotid artery injury (74). Besides, VCAM-1 participates in leukocyte exudation, and ICAM-1-siRNA delivered *via* ultrasound microbubbles can reportedly effectively retard arterial intimal hyperplasia (75). Intriguingly, Huang et al. found that ICAM-1 antibodies could inhibit the replication of VSMCs (76). Taken together, chemokines, adhesion factors and inflammatory cytokines orchestrate the phenotypic transformation of VSMCs and the development of vascular intimal hyperplasia.

### Discovery, Characteristics and Functions of miRNAs

In 1993, researchers identified a non-coding RNA that could repress the expression of lin-14 and thus affect nematode development (77). Subsequently, let-7, the second miRNA, was also discovered in nematodes (78). Since then, more and more miRNAs have been identified in human, animal, and plant genomes, and scholars have unveiled that miRNAs are widely present in eukaryotes and participate in the biological effects. miRNAs represent endogenous and non-coding RNAs with independent transcription units, containing about 22 nucleotides that function mainly by recognizing and binding to the 3’ untranslated region (3’UTR) of the messenger RNA of their targets *via* complementary base pairing. miRNAs are evolutionarily conserved, homologous and tissue-specific in species (79). The base pair between nucleotides 2 to 8 at the 5’-end of miRNAs serves as the most central and conservative region, called the seed sequence. The seed sequence is the core region that binds to the 3’ UTR of the targets (80). The slice and maturation of miRNA undergo three main processes: the generation of primary miRNA (pri-miRNA), the generation of precursor miRNA (pre-miRNA) and its transportation out of the nucleus, and the maturation of miRNAs. It has been established that more than 2000 miRNAs are encoded by the human genome, regulating one-third of human genes (81). In addition, evidence from studies indicates some atypical examples of miRNAs functionality and localization, including interactions with proteins beyond the argonaute family and transcriptional regulation in the nucleus and in mitochondria (82). It further expands the importance of its role in regulatory function of organism. miRNAs engage in many biological processes, including growth, aging, angiogenesis, immune regulation, metabolism, cell proliferation, apoptosis, cell migration, tumorigenesis, metastasis, and drug resistance.

### Role of miRNAs in the Phenotypic Switch of Vascular Smooth Muscle Cells (VSMCs) in Intimal Hyperplasia of Vein Graft

miRNAs are widely expressed in the blood vascular system and regulate vascular cell growth and function. Knock-out of dicer (an enzyme essential for miRNAs maturation) showed arrest of embryogenesis due to the inability to generate normal blood vessels, suggesting that miRNAs may be essential for angiogenesis (83). miR-145-5p is the most abundant miRNA in VSMCs, and it promotes the differentiation of mesenchymal stem cells (MSCs) to VSMCs through the Kruppl-like factor 4 (KLF4) pathway (84). Antagonizing miR-1 expression could lead into VSMCs-specific markers and inhibit the differentiation of embryonic stem cell-derived VSMCs *via* the KLF4 pathway (85). Each miRNA can regulate the expression and function of multiple targets *via* the same signal pathway. Indeed, it should be borne in mind that a miRNA could regulate many targets. Currently, many miRNAs are involved in the regulation of VSMCs. Many miRNAs with regulatory effects on the function and phenotype of vascular VSMCs have been identified in recent years. miR-1, miR-15b/16-5p, miR-21, miR-22, miR-23b, miR-34a, miR-125b, miR-126-3p, miR-132, miR-133(miR-133a-1/miR-133a-2), miR-143-5p/145-5p, miR-195, miR-214, miR-223, miR-548f-5p, miR-638, miR-663, and miR-1298-5p miRNAs help maintain the differentiated phenotype of VSMCs and curb the proliferation and migration of VSMCs (84–102). Among above-mentioned miRNAs, miR-23b, miR-25, miR-125, miR-143-5p/145-5p, miR-221/222, miR-214, miR-638 and miR-663 can mediate the function and phenotype switch of VSMCs induced by PDGF-BB, and all of them participate in the regulation of intimal hyperplasia in injured vessels.

The etiologies, factors and molecular mechanisms described above do not exist alone and often interact to influence VSMCs functions in vessel disease. Interesting, the study by Zahedi et al. shows that dicer activity controls neointimal hyperplasia by reducing VSMCs proliferation after vascular injury. The levels of miR-147-3p, miR-143-3p, miR-100-5p, miR-99a-5p, and miR-27a-3p were most significantly reduced in dicer knockout mice, upon further study, Zahedi et al. found that these miRNAs may be involved in this process of VSMCs phenotypic regulation by influencing inflammation-induced growth factor signaling (103). It is suggests that general suppression of miRNA synthesis and growth factor signaling play a part role in regulation of VSMCs phenotype. The study by Sun et al. indicates that miR-133a-3p is reduced significancely in carotid artery ligation-induced vascular injury, Salusin-β is upregulated, MMP9 expression and reactive oxygen species (ROS) production is increased, promotes VSMCs migration and neointima formation in response to vascular injury (104). It has been shown that ischemic-hypoxic injury with ROS production and oxidative stress is an important cause of vascular remodeling (105). Previous studies have showed that miR-21, miR-24 miR-31-5p and miR-210 regulates ischemia/hypoxia stress-induced VSMCs functions in vascular disease (105–108). Differences in miRNA expression patterns as a function of hemodynamic forces have been detected, which is named the term “mechano-miRNAs.” miRNAs such as miR-33, miR-126 and miR-143-5p/145-5p are counted amongst those mechano-miRNAs (8, 45, 92). In particular, miR-126 is an endothelial enriched miRNA, Jansen et al. found that the transfer of miR-126-3p mediated by endothelial particles into recipient VSMCs could inhibit the its proliferation, migration and subsequent neointima formation of injuried artery (92). The study by Santovito et al. showed that, under high shear stress, the nuclear import of miR-126-5p is reduced, promotes ECs apoptosis and exacerbates atherosclerosis (109). On the one hand, it indicates that mechano-miRNAs is vital in phenotype transformation of VSMCs. On the other hand, it reveals a non-canonical mechanism by which miRNAs including miR-126-5p may modulate protein function in VSMCs, as already proven in ECs for caspase-3 (109). Moreover, miR-126-5p has been shown to promote the contractile phenotype of VSMCs (110). It is well known that miR-143-5p/145-5p has been confirmed to control VSMCs phenotype in a large number of studies. It is called the regulator of VSMCs. The study by Climent et al. showed that vessel stress triggers miR-143-5p/145-5p transfer from VSMCs to their neighboring ECs to modulate the angiogenesis and vascular homeostasis (111). Hergenreider et al. research indicated that extracellular vesicles secreted by shear-stress-stimulated ECs are enriched in miR-143-5p/145-5p and regulate target gene expression in co-cultured VSMCs (112). These interesting studies suggest that some miRNAs such as miR-143-5p, miR-145-5p act as communication molecules between VSMCs and ECs exposure to shear stress. It also suggests that some miRNAs plays an important role in phenotypic regulation of VSMCs causing by non-physiological shear stress.

In brief, miRNAs play a crucial role in the phenotype transformation of VSMCs, and in-depth research on their functions, targets and interaction networks can help understand proliferative diseases of VSMCs such as intimal hyperplasia after vessel injury, intimal hyperplasia in vein graft after CABG surgery, and atherosclerosis from a new perspective, offering therapies for prevention and treatment. The widely acknowledged causes of intimal hyperplasia in vascular disease are injury from surgery, ischemic-hypoxic injury, and stress alterations, which are associated with dysfunction and phenotypic switch of VSMCs. Intimal hyperplasia caused by VSMCs phenotype and function change results from the interplay of multiple factors. At present, the literature reports are very rare that the regulation of miRNAs on inflammatory factors, metalloproteinases, extracellular matrix, tissue factor and growth factor influencing the phenotypic switch of venous VSMCs in vein graft. The further research is still required.

**FIGURE 2:**
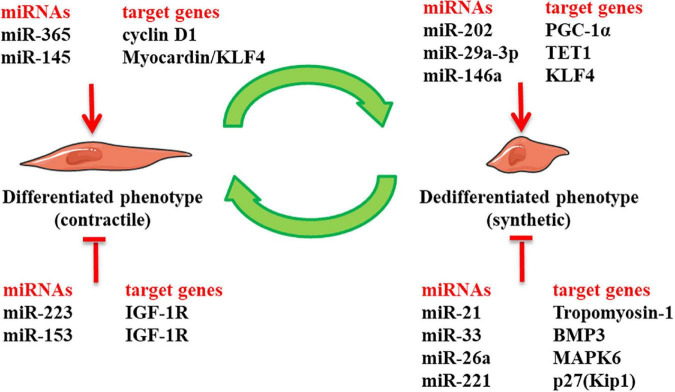
The role of miRNAs in regulating phenotypic switch of venous smooth muscle cells (SMCs) in intimal hyperplasia of vein graft: it presents miRNAs that have been identified bearing regulatory effects on the phenotype of venous vascular smooth muscle cells (VSMCs) in intimal hyperplasia of vein graft. Klf4, Kruppl-like factor 4; IGF-1R, insulin-like growth factor-1 receptor; PCG-1α, peroxisome proliferator-activated-receptor-γ coactivator-1α; TET1, Ten-eleven translocation methylcytosine dioxygenase 1; BMP3, bone morphogenetic protein 3; MAPK6, mitogen-Activated Protein Kinase 6; p27(Kip1), p27KIP1 gene.

It is widely acknowledged that miRNAs are essential for regulating the proliferation and differentiation of VSMCs during embryogenesis (113). Many cardiovascular diseases are relevant to the abnormal expression and function of miRNAs (114). As shown in Figure 2, miRNAs exhibit regulatory effects on the function and phenotype of venous SMCs (115–121). Furthermore, many studies have shown that miRNA regulation of phenotypic transformation of VSMCs is vital for intimal hyperplasia in vein grafts. miR-21 and miR-145 are the most plentiful miRNAs expressed on the vessels. Studies in rats have shown that miR-21 is significantly upregulated after vessel damage with decreased endogenous c-Ski and increased VSMC proliferation (87). In addition, the Phosphatase and Tensin Homolog (PTEN) gene serves as a target for miR-21, and overexpression of miR-21 inactivates PTEN, minimizing apoptosis and maximizing the proliferation of VSMCs (105). Interestingly, some researchers have documented a significantly elevated miR-21 level in intimal hyperplasia of vein graft in mouse and porcine models, also observed in human decaying grafted veins. Focal knockdown of miR-21 in vein grafts could limit the number of VSMCs in the nascent intima and attenuate intimal hyperplasia (116). In this regard, miR-21 has been documented to regulate the phenotype of VSMCs, involving the occurrence of intimal hyperplasia in grafted veins. Likewise, miR-145 propels the expression of differentiation genes to maintain the differentiated state of VSMCs *via* the TGF-β signaling pathway (117). miR-145 fosters the expression of SM22α that a specific marker of differentiated phenotype, and inhibits the phenotype transformation of VSMCs and their ability to proliferate and migrate by curbing the expression of the KLF4 transcription factor (95). The overexpression of miR-145 impedes the phenotypic transformation of VSMCs and ameliorates the intimal hyperplasia of vein graft in a rabbit model (118). The above-mentioned studies conclude that miR-21 has a pro-VSMCs dedifferentiation effect, and miR-145 can stimulate cell differentiation and maintain the differentiated phenotype of venous SMCs. The vasculature represents a typical mechanical system comprised of VSMCs, which are the primary cells of the vessel wall and are vital for enduring pressure changes and maintaining vascular functional homeostasis. Exposure of venous SMCs to a 10% 1.25 Hz cyclic stretch yielded waning expression of miR-33 and a waxing proliferation of venous SMCs. This phenomenon was verified in rat models; the downregulation of miR-33 and upregulation of phosphorylated smad2/5 upregulated VSMCs replication and exacerbated intimal hyperplasia in vein grafts. Overexpression of miR-33 was found to remarkably hinder the formation of intimal hyperplasia in vein graft (8). Interestingly, difference from only one miR-33 isoform in rodents which is conserved with human miR-33a, miR-33 exist two isoforms in humans: miR-33a and miR-33b. Therefore, we should pay attention to the different functions of the miRNAs ‘ isoforms in different species and cells. Moreover, Wang et al. found that after Matrigel-127 mixed adenovirus transfection to silence miR-221 in rat models, the proliferation of venous SMCs was reduced by approximately 20% with improved blood flow of vein graft (119). Using the above method to overexpress miR-365, Cao et al. found that it downregulated the expression of G1/S-specific cyclin D1 and amplified the expression of differentiation-type markers in VSMCs, thereby inhibiting intimal hyperplasia in the grafted vein (120). Intriguingly, miR-16-5p was downregulated in rat vein grafts with alterations in phenotypic markers, and miR-16-5p overexpression suppressed intimal hyperplasia. Mechanistically, miR-16-5p could disrupt the expression of zyxin, a mechanotransducer of biological signals, *via* binding to the 3’UTR of the zyxin- mRNA, which inhibited the proliferation and migration of cultured VSMCs by preventing the switch from a contractile to a synthetic phenotype. Our study implies that miR-16-5p is a potential therapeutic target for combating intimal hyperplasia in vein grafts (121). miRNAs can be used as targets for preventing VSMCs from switching phenotype and preventing intimal hyperplasia after vein grafting.

In conclusion, miRNAs are pronounced regulators in the biological processes of differentiation, proliferation and migration of VSMCs, formulate a phenotype switch of VSMCs and influence the occurrence of intimal hyperplasia in grafted veins, which broaden the therapeutic horizons for vein graft restenosis in clinical practice.

### Prospect

Restenosis induced by intimal hyperplasia of the graft veins after CABG is the main factor affecting surgery outcomes in CAD patients. In the process of surgical procedures, there is sufficient time for genetic manipulation, so that controlling molecular alterations in the initial stage of injury, making it possible to prevent intimal hyperplasia of the graft veins. Moreover, focal gene interventions during the procedure shun the side effects of systemic administration, which is ideal for prophylaxis of vein graft degeneration. VSMCs are one of the most important cells of the vessel wall, and the phenotypic transformation of VSMCs plays an important part in the mechanism of vein graft intimal hyperplasia-induced atherosclerosis. miRNAs are well-studied non-coding RNAs with independent transcription units and stable expression, involved in many biological processes. miRNAs have been discovered to regulate the switch of VSMC phenotypes. By intervening and maintaining the differentiated phenotype of VSMCs, miRNAs are poised to provide a novel strategy of local gene therapy for the prophylaxis and treatment of vein graft failure and other cardiovascular diseases. miRNAs represents a promising technique for silencing genes that cause above-mentioned intimal hyperplasia in vein graft after CABG surgery as well as other cardiovascular diseases caused by phenotypic switch of VSMCs, such as aterial intimal hyperplasia after vessel injury, pulmonary hypertension, hypertension and atherosclerosis.

miRNAs represents a promising technique for silencing genes that cause above-mentioned cardiovascular diseases. The successful delivery of miRNAs to the vascular wall faces multiple challenges (122). Firstly, these challenges include cell specificity, targeted delivery of miRNAs, anatomical tissue barriers. Secondly, there is no report of studies to translate findings of miRNAs in proliferative cardiovascular diseases from rodent models into human studies. This may be due to face risks of toxicity and side effects due to a wide range of target genes and cells. In addition, the selection of the target genes has become a difficult problem. There are considerable number of miRNAs that regulate the proliferative of VSMCs in cardiovascular diseases. Which miRNA is the most effective molecule to regulate the phenotype of VSMCs or whether multiple genes are required to work together in the process of phenotypic switch of VSMCs, and what is the detailed and specific mechanism and so on, a further research is still required.

The role of miRNAs in cardiovascular diseases is a rising research filed. Elucidating the regulatory mechanism of miRNA in the organism can help understand proliferative cardiovascular diseases of VSMCs such as intimal hyperplasia after vessel injury, intimal hyperplasia in vein graft after CABG surgery, pulmonary hypertension, hypertension and atherosclerosis from a new perspective, offering miRNA therapy.

## Author Contributions

All authors listed have made a substantial, direct, and intellectual contribution to the work, and approved it for publication.

## Conflict of Interest

The authors declare that the research was conducted in the absence of any commercial or financial relationships that could be construed as a potential conflict of interest.

## Publisher’s Note

All claims expressed in this article are solely those of the authors and do not necessarily represent those of their affiliated organizations, or those of the publisher, the editors and the reviewers. Any product that may be evaluated in this article, or claim that may be made by its manufacturer, is not guaranteed or endorsed by the publisher.
